# Sugary Soda Consumption and Albuminuria: Results from the National Health and Nutrition Examination Survey, 1999–2004

**DOI:** 10.1371/journal.pone.0003431

**Published:** 2008-10-17

**Authors:** David A. Shoham, Ramon Durazo-Arvizu, Holly Kramer, Amy Luke, Suma Vupputuri, Abhijit Kshirsagar, Richard S. Cooper

**Affiliations:** 1 Department of Preventive Medicine and Epidemiology, Stritch School of Medicine, Loyola University Chicago, Maywood, Illinois, United States of America; 2 Division of Nephrology, Department of Medicine, Loyola University Medical Center, Maywood, Illinois, United States of America; 3 UNC Kidney Center, University of North Carolina, Chapel Hill, North Carolina, United States of America; 4 Department of Epidemiology, University of North Carolina, Chapel Hill, North Carolina, United States of America; 5 Center for Health Research, Kaiser Permanente, Atlanta, Georgia, United States of America; L' Istituto di Biomedicina ed Immunologia Molecolare, Consiglio Nazionale delle Ricerche, Italy

## Abstract

**Background:**

End-stage renal disease rates rose following widespread introduction of high fructose corn syrup in the American diet, supporting speculation that fructose harms the kidney. Sugar-sweetened soda is a primary source of fructose. We therefore hypothesized that sugary soda consumption was associated with albuminuria, a sensitive marker for kidney disease.

**Methodology/Principal Findings:**

Design was a cross-sectional analysis. Data were drawn from the National Health and Nutrition Examination Survey (NHANES), 1999–2004. The setting was a representative United States population sample. Participants included adults 20 years and older with no history of diabetes mellitus (n = 12,601); after exclusions for missing outcome and covariate information (n = 3,243), the analysis dataset consisted of 9,358 subjects. Exposure was consumption of two or more sugary soft drinks, based on 24-hour dietary recall. The main outcome measure was Albuminuria, defined by albumin to creatinine ratio cutpoints of >17 mg/g (males) and >25 mg/g (females). Logistic regression adjusted for confounders (diet soda, age, race-ethnicity, gender, poverty). Interactions between age, race-ethnicity, gender, and overweight-obesity were explored. Further analysis adjusted for potential mediators: energy intake, basal metabolic rate, obesity, hypertension, lipids, serum uric acid, smoking, energy expenditure, and glycohemoglobin. Alternative soda intake definitions and cola consumption were employed.

**Results:**

Weighted albuminuria prevalence was 11%, and 17% consumed 2+ sugary soft drinks/day. The confounder-adjusted odds ratio for sugary soda was 1.40 (95% confidence interval: 1.13, 1.74). Associations were modified by gender (p = 0.008) and overweight-obesity (p = 0.014). Among women, the OR was 1.86 (95% CI: 1.37, 2.53); the OR among males was not significant. In the group with body mass under 25 kg/m^2^, OR = 2.15 (95% confidence interval: 1.42, 3.25). Adjustment for potential mediators and use of alternative definitions of albuminuria and soda consumption did not appreciably change results. Diet sodas were not associated with albuminuria.

**Conclusions:**

Findings suggest that sugary soda consumption may be associated with kidney damage, although moderate consumption of 1 or fewer sodas does not appear to be harmful. Additional studies are needed to assess whether HFCS itself, overall excess intake of sugar, or unmeasured lifestyle and confounding factors are responsible.

## Introduction

The rising incidence of End Stage Renal Disease (ESRD)in the United States over the past three decades is only partially attributable to increasing prevalence of recognized risk factors, most prominently diabetes mellitus [1]. The etiology of diabetes is multi-causal, but increased energy intake and a diet rich in high-glycemic-index foods are two likely culprits. Trends in the U.S. diet further contribute to obesity, which has recently emerged as an independent risk factor for kidney disease [2], [3], [4], [5]. Trends in diabetes, obesity and kidney disease have all followed the introduction of high fructose corn syrup (HFCS) in the American diet [6], [7]. In the United States, highly caloric carbonated soft drinks are often sweetened with HFCS [8], which makes soda inexpensive to produce. While sugary sodas may be sold cheaply as a food item and yield a profit, they provide little satiety [9].

Kidney disease exacts an extensive human and economic price through both ESRD and greatly elevated cardiovascular disease risk [10], [11], [12]. Chronic kidney disease (CKD) is assessed in a number of ways. In epidemiologic studies, estimated glomerular filtration rate (GFR) and albuminuria are most commonly used [13]. Albuminuria has the advantage of being a sensitive marker for early kidney damage, and is prevalent in over 10% of the U.S. adult population [14]. In the general population, albuminuria predicts incident kidney failure [15] and cardiovascular disease, even at levels considered “normal” [16], [17], [18].

Three mechanisms may relate soda consumption to albuminuria. First, as noted, the low cost of HFCS makes it profitable for manufacturers and inexpensive for consumers, encouraging consumption of large amounts of soda. Over-consumption of sugars in any form may lead to the development of subclinical diabetes, which may eventually manifest as kidney damage years before clinical diagnosis and the onset of overt diabetes [19]. Obesity, also related to CKD, may be another consequence of high levels of sugary soda consumption [9]. Second, fructose itself may cause kidney damage, perhaps mediated by uric acid [6]. Third, other ingredients in soda, such as phosphorus in dark cola, may lead directly to kidney damage [20].

Therefore, we sought to determine whether sugary soft drink consumption was associated with albuminuria, a sensitive marker of early kidney damage, in the general population.

## Methods

Data were drawn from the U.S. population-based National Health and Nutrition Examination Survey (NHANES), 1999–2004. The NHANES is a complex, nationally representative sample of the entire non-institutionalized United States population, now conducted in continuous bi-annual waves by the Centers for Disease Control and Prevention [21]. We included only those subjects 20 years and older without self-reported history of diabetes and not missing diabetes status (n = 12,601). Exclusions were made for those missing albuminuria outcome (n = 1,193), missing dietary recall information (n = 693), and/or missing confounder data (n = 2,020). A total of 3,243 subjects were thus excluded, yielding an analysis dataset of 9,358 subjects with complete data (74.3% of the non-diabetic cohort age 20 and older). The current study was approved by the Loyola University Chicago Stritch School of Medicine Institutional Review Board.

Albuminuria, the outcome variable in the primary analyses, was defined by sex-specific albumin to creatinine ratio (ACR) cutpoints of ≥17 mg/g in males and ≥25 mg/g in females; following Mattix and colleagues, lower sex-specific cutpoints for males were chosen based on their higher urinary creatinine concentrations for any given level of albumin excretion [22]. GFR was estimated using the abbreviated Modification of Diet in Renal Disease (MDRD) estimating equation [23]. While GFR was compared between albuminuria cases and non-cases, it was not the focus of this study and was only included in secondary analyses. According to available information, missing subjects had +1.20 mg/L higher urinary albumin concentration (p<0.001 on the log scale). Missing subjects were on average 1.6 years younger than non-missing subjects (p<0.001) and had a body mass index that was an average of 0.50 kg/m^2^ lower (p = 0.008).

**Figure 1:**
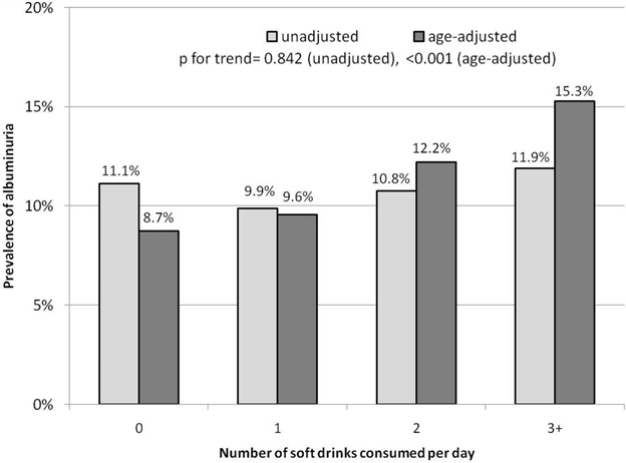
Prevalence of albuminuria among NHANES 1999–2004 non-diabetics age 20 and over, unadjusted and adjusted for age, according to sugary soft drink consumption.

All analyses were weighted by mobile examination clinic (MEC) weights to account for the complex survey design, and analyzed using STATA version 9.2 *svy* commands (STATA Corp., College Station, TX) according to NHANES analytical guidelines [24]. Soda consumption was obtained from 24 hour dietary recall (USDA Food Codes 92400000 through 92411610). Sugar-sweetened and diet sodas were separately dichotomized as 0–1 (reference) vs. 2 or more drinks per day. The choice of this cutpoint was based on preliminary analyses, which showed the association between sugary drinks and albuminuria was similar for 0 and 1 drinks per day (8.7 and 9.6%, respectively; see Figure 1). Unadjusted logistic regression was first conducted for associations with albuminuria. Next, Model 1 adjusted for diet soda consumption (2 or more, vs. 0–1 per day), age, non-Hispanic black race or Hispanic ethnicity (reference: non-Hispanic white), gender, and poverty; following Martins and colleagues, we defined poverty as falling below twice the U.S. federal poverty guidelines (for a non-elderly household of 2 in the year 2000, this was an annual household income under $23,180) [25]. Race-ethnicity was by self report, according to pre-set categories. A further analysis (Model 2) included several variables assumed to be mediators of any association, to see if any association with soda consumption persisted. Model 2 included all of the variables in Model 1, plus hypertension, glycohemoglobin A1C, total caloric consumption, smoking (current vs. former or never), obesity, total cholesterol, and a summary measure of physical activity (the sum of all reported physical activity weighted by metabolic equivalent level or MET for each activity, expressed as total MET-minutes; see http://www.cdc.gov/nchs/data/nhanes/frequency/paqiaf_doc.pdf for more detail). Hypertension was a dichotomous variable defined according to Seventh Joint National Committee guidelines as systolic blood pressure ≥140, diastolic blood pressure ≥90, self-reported history of hypertension and/or use of antihypertensive medication [26].

In order to investigate effect measure modification of associations, interaction terms were introduced into the model between consuming 2 or more sugary sodas and indicator variables for the following: gender (male or female); self-reported race-ethnicity by pre-defined categories (Non-Hispanic White, reference; Non-Hispanic Black; or Hispanic of any race); age; and by overweight/obesity category (≥17.5 & <25, “low to normal weight”; ≥25 & <30, “overweight”; and ≥30 kg/m^2^, “obese”). Significant interaction was defined at the p = 0.10 level, indicating effect measure modification and warranting subgroup analyses. Because serum uric acid levels have been associated with fructose consumption [6], [27], we assessed interaction with serum uric acid (as a continuous variable) and with hyperuricemia (categorically defined as the highest quartile of serum uric acid, 6.3 mg/dL or higher). We then investigated whether any associations between sugary soft drinks and albuminuria differed between sugar-sweetened or diet colas and non-colas.

Several sensitivity analyses were conducted. Because 24-hour recall is an imperfect measure of caloric intake and has known biases by BMI [28], [29], [30], [31], we compared the consumption pattern of 2+ sodas per day as a function of both BMI category and quartile of energy intake. We calculated Pearson χ^2^ p-values for row (BMI category within energy intake quartile) and column (energy intake quartile within BMI categories) differences in proportion in sugary soda intake; if there are significant biases by BMI category, then χ^2^ p-values for rows should be significant. We expect column χ^2^ p-values to be significant, since sugary soda consumption should rise with increasing total daily energy intake. Table 1 does not indicate any systematic biases by BMI category, except for the 2^nd^ quartile of energy intake (p = 0.035); nevertheless, the pattern is for greater sugary soda consumption to be reported among underweight and obese subjects, which is not consistent with the prediction that underreporting would be greatest in the obese subjects [31], [32].

**Table 1:** Proportion consuming 2+ sugary sodas (cells) by quartile of energy intake (rows) and body mass index (BMI) category (columns).

Quartile of energy intake (kcals/day)	BMI Category				χ^2^ *p-value (row differences)*
	Underweight (17.5–20)	Normal (20–25)	Overweight (25–30)	Obese (30+)	
1 (<1350.0 kcal/day)	6.4	7.2	5.3	5.9	*0.741*
2 (1350.0–1859.8)	17.7	10.4	10.4	14.9	*0.035*
3 (1860.0–2510.1)	20.8	15.7	15.5	14.7	*0.431*
4 (>2510.1)	29.4	25.1	24.1	30.1	*0.109*
χ^2^ *p-value (column differences)*	*0.008*	*<0.001*	*<0.001*	*<0.001*	

Note: “Column differences” χ^2^ p-value assess whether sugary soda consumption differs by level of caloric consumption within each BMI category. “Row differences” χ^2^ p-value assess whether sugary soda consumption differs by BMI category within each level of energy intake.

In order to determine that the results of Models 1 and 2 were not due to macroalbuminuria, we excluded those subjects with ACR≥300 mg/g (n = 120, 1.3% of the analysis dataset). We then used linear regression with log-transformed urinary albumin as the dependent variable, and soda consumption (dichotomized as above) as the main exposure of interest, adjusting for age, race, ethnicity, gender, poverty, and the reciprocal of urinary creatinine; we modeled the log of albumin concentration, rather than ACR, as the dependent variable because the latter approach may introduce spurious correlation [33]. Next, a logistic model similar to Model 1 was used, but rather than categorizing sugary soda consumption at the cutpoint of 2+ vs. 0–1, we instead defined exposure as highest quintile group of sugary soda consumption (by grams of sugar intake from soda). Further models adjusted for total energy intake (in total kilocalories), alone or in combination with basal metabolic rate (estimated using the Harris-Benedict equation, [34]). We then used continuous eGFR in a linear model, and dichotomized eGFR in logistic models; the dichotomous models employed both the KDOQI guideline for Stage 3 CKD (eGFR<60 ml/min/1.73 m^2^), and a cutpoint with greater specificity (eGFR <45 ml/min/1.73 m^2^) as suggested by Go [35] and Shoham [36].

## Results

The study population was distributed evenly by gender and was predominantly non-Hispanic White (55.6%); due to oversampling, 25.5% of the study population was African-American, and 33% Hispanic. Summary statistics, overall and by albuminuria status, are reported in Table 2 (note that results reported in the tables take into account the complex survey design, including oversampling, and extrapolate back to the population from which they are drawn). The mean age of the study population was 45.1 years. Seventeen percent reported consuming two or more sugary sodas a day, while 11% had elevated albuminuria (n = 1326; the prevalence is extrapolated back to the general population). Participants with albuminuria were more likely to be African-American, obese, hypertensive, be physically inactive, or live below twice the poverty line than participants without albuminuria. Mean ACR, triglyceride and total cholesterol level, BMI and blood pressures were higher, and mean GFR lower, in albuminuria cases compared to non-cases. There were no crude differences in sugary soda consumption between cases and non-cases. However, younger participants (ages 20–29) were more likely than older ones (ages 65+) to drink sugary sodas (27.5% vs. 5.3%; p for trend<0.001), yet the younger subjects are also less likely to have albuminuria. Figure 1 shows that there is no crude relationship between sugary sodas and albuminuria (p for trend = 0.84), while age adjustment reveals a clear, positive dose-response relationship (p for trend<0.001).

**Table 2:** Distribution of attributes, National Health and Nutrition Examination Survey 1999–2004, overall and by albuminuria status.

	Overall	No Albuminuria	Albuminuria	p-value
	n = 9358	n = 8032	n = 1326	
**Mean (standard error)**				
Age (years)	45.1 (0.5)	44.0 (0.5)	54.6 (1.2)	<0.001
Number of Sugary Drinks/Day	0.67 (0.04)	0.68 (0.04)	0.6 (0.06)	0.83
Colas	0.38 (0.02)	0.38 (0.02)	0.33 (0.04)	0.11
Non-colas	0.29 (0.02)	0.29 (0.02)	0.27 (0.03)	0.208
Number of Diet Drinks/Day	0.27 (0.03)	0.28 (0.03)	0.18 (0.04)	0.01
Poverty-Income Ratio	3.2 (0.1)	3.2 (0.1)	2.7 (0.1)	<0.001
Systolic Blood Pressure (mm Hg)	121.7 (0.5)	120.3 (0.5)	135.2 (1.2)	<0.001
Diastolic Blood Pressure (mm Hg)	72.8 (0.5)	72.6 (0.4)	74.6 (0.9)	0.01
Body Mass Index (kg/m^2^)	27.8 (0.2)	27.7 (0.2)	28.1 (0.6)	0.03
Serum glycohemoglobin (mg/dL)	5.32 (0.01)	5.30 (0.01)	5.51 (0.05)	<0.001
Serum uric acid (mg/dL)	5.42 (0.03)	5.37 (0.03)	5.85 (0.09)	<0.001
Serum Total Cholesterol (mg/dL)	202.4 (0.7)	202.0 (0.7)	206.1 (2.0)	0.026
Serum Triglyceride (mg/dL)†	144.9 (3.1)	141.5 (2.9)	175.1 (12.8)	0.009
Reported Energy Intake (1000 kcal/day)	2.27 (0.02)	2.30 (0.03)	2.07 (0.05)	<0.001
Basal Metabolic Rate (1000 kcal/day)	1.49 (0.01)	1.50 (0.01)	1.43 (0.03)	0.01
Glomerular Filtration Rate (ml/min/1.73 m^2^)	100.1 (0.6)	100.7 (0.6)	95.5 (1.1)	<0.001
Albumin-to-Creatinine Ratio (mg/g)	22.5 (4.4)	6.5 (0.1)	171.5 (43.3)	n/a
**Proportions (%)**				
African-American	10.2	9.9	12.7	0.004
Hispanic	12.8	13.2	12.5	0.50
Male	50.4	50.0	51.3	0.47
Hypertension	25.3	23.3	40.2	<0.001
Current Smoker	25.0	25.0	25.8	0.57
Overweight	35.7	36.5	31.1	0.001
Obese	29.7	29.0	35.6	0.001
No moderate-vigorous physical activity	33.6	32.4	44.6	<0.001
Below 2× Poverty	32.3	30.9	40.0	<0.001
2+ Sugary Sodas/ Day	16.8	16.8	17.5	0.60

P values use t-test for differences in means, and Wald chi-square test for proportions.

* Note that values in the table are weighted to take into account the complex survey design; however, the counts of number of subjects with (n = 1326) and without albuminuria (n = 8032) are not weighted for the survey design.

† Triglyceride levels available only for the subset (n = 4457) with fasting morning blood draw.

Using the “basic” Model 1, adjusting for consumption of 2 or more diet sodas per day, age, race-ethnicity, gender, and living below twice the poverty level (Table 3), there was 40% increased odds of having albuminuria associated with consuming two or more sugary sodas per day (odds ratio [OR]: 1.40; 95% confidence interval [CI]: 1.13, 1.74). Diet soda consumption was not associated with albuminuria. Model 2 (Table 3), which added several variables thought to be confounders and mediators of the sugary soda and albuminuria association, yielded similar results to Model 1.

**Table 3:** Unadjusted and adjusted Odds Ratios (OR) and 95% Confidence Intervals (95%CI) for albuminuria.

	Unadjusted associations	Model 1: Adjusted for confounders	Model 2: Model 1+mediators
	OR (95% CI)	OR (95% CI)	OR (95% CI)
2+ sugary drinks/day (vs. 0–1)	1.05 (0.85, 1.29)	1.40 (1.13, 1.74)	1.33 (1.03, 1.72)
2+ diet drinks/day (vs. 0–1)	0.74 (0.51, 1.07)	0.94 (0.64, 1.39)	0.97 (0.63, 1.47)
Age (per 10 year increase)	1.39 (1.33, 1.45)	1.43 (1.37, 1.49)	1.34 (1.25, 1.43)
African American (vs. Non-Hispanic White)	1.31 (1.08, 1.60)	1.40 (1.13, 1.74)	1.23 (0.98, 1.56)
Hispanic (vs. Non-Hispanic White)	0.98 (0.81, 1.18)	1.07 (0.87, 1.32)	1.16 (0.92, 1.46)
Male (vs. Female)	1.05 (0.92, 1.18)	1.18 (1.03, 1.36)	1.12 (0.94, 1.35)
Below 2× Poverty Line (vs. Above)	1.65 (1.33, 2.05)	1.77 (1.47, 2.12)	1.68 (1.36, 2.09)
Energy intake (1000 kcal difference)	0.82 (0.77, 0.88)		0.92 (0.84, 1.01)
Obesity (vs. Normal or Overweight)	1.36 (1.14, 1.61)		1.04 (0.88, 1.24)
Total Cholesterol (per 100 mg/dL increase)	1.26 (1.04, 1.53)		0.90 (0.73, 1.11)
Hypertension (all definitions)	2.59 (2.16, 3.11)		1.59 (1.29, 1.96)
Uricemia (uric acid >6.8 mg/dL)	3.70 (2.59, 5.28)		1.60 (1.28, 2.01)
Glycohemoglobin (per mg/dL)	1.85 (1.63, 2.11)		1.39 (1.25, 1.53)
Current smoker (vs. former/never)	1.05 (0.88, 1.21)		1.25 (1.02, 1.52)
Physical Activity (per 1000 METS-min)	0.98 (0.97,0.99)		1.00 (0.99, 1.01)

There was no interaction between sugary soda consumption and age (p = 0.14), black or Hispanic race-ethnicity (p = 0.88 and 0.33, respectively), continuous serum uric acid level (p = 0.52), or hyperuricemia (p = 0.35). There was a significant negative interaction between sugary soda consumption and being overweight (p = 0.005) or obese (p = 0.02) and with male gender (p = 0.008), indicating the need for subgroup analyses. Among women, the OR was 1.86 (95% CI: 1.37, 2.53). In the group with low-to-optimal body weight (BMI 17.5–25 kg.m^2^), consumers of 2 or more sugary sodas had 2.15 times the odds of albuminuria as those who consumed 0–1 sugary drinks (95% CI: 1.42, 3.25). Associations in other subgroups were weak and imprecise.

**Figure 2:**
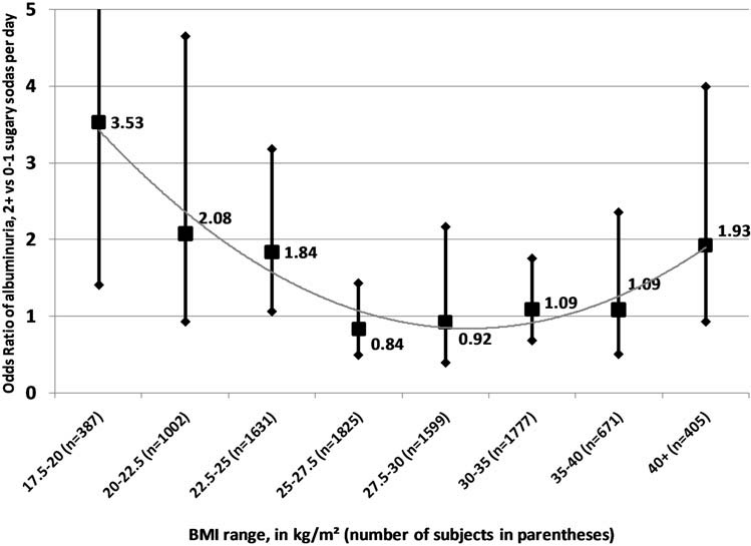
Adjusted Odds Ratios (aORs) comparing albuminuria among consumers of 2+ vs. 0–1 sugary soft drinks per day, stratified by body mass index (BMI) category. Trend line shows a quadratic model fit to the aORs; vertical lines represent 95% Confidence Intervals. The aORs are adjusted for age, race, ethnicity, and poverty status, but not BMI. BMI is used only as a stratification variable. Figure excludes subjects with BMI<17.5 kg/m^2^ (n = 61).

**Figure 3:**
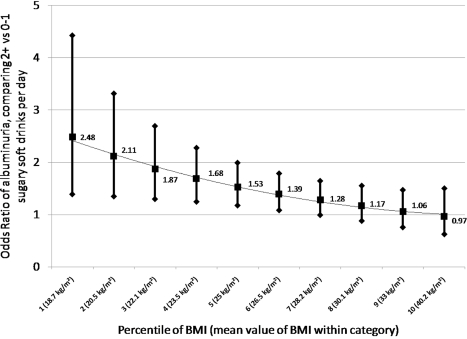
Adjusted Odds Ratios (aORs) comparing albuminuria among consumers of 2+ vs. 0–1 sugary soft drinks per day, according to interaction with body mass index (BMI). Adjusted for diet soda consumption, age, race, ethnicity, poverty status, BMI, and BMI-squared.

The finding of negative interaction between obesity and sugary soda consumption was unexpected, warranting further investigated of the relationship between BMI, soda consumption, and albuminuria. We conducted a stratified analysis, fitting Model 1 within categories of underweight, optimal weight, overweight, and obesity. Because the relationship between sugary soda consumption and albuminuria appears to be modified in a quadratic manner (Figure 2), we added to the variables in Model 1 the following: BMI, BMI-squared, and interaction terms between soda consumption and continuous BMI and BMI-squared (respective p-values for interaction: 0.04 and 0.10). Due to the interaction terms, there is no true main effect for sugary soda consumption in this model. The results are reported in Figure 3, which shows that the strongest association between sugary soda consumption and albuminuria was at the low end of BMI: at 18.7 kg/m^2^ (the midpoint of the lowest decile of BMI), the odds ratio was 2.48 (95% CI: 1.39, 4.42), while at a BMI of 40.2 (the midpoint of the highest decile), the OR was 0.97 (95% CI: 0.62, 1.50). We conclude that sugary sodas are most strongly associated with albuminuria at the low end of body weight.

We then categorized sugary soda consumption as sugary cola, sugary non-cola, diet cola, and diet non-cola, with the reference being all other beverages. The strongest associations appeared for consuming 2 or more sugary non-colas per day (OR = 1.77; 95%CI: 1.28, 2.45). Sugary cola, diet cola, and non-cola consumption were not associated with albuminuria. Adjusting for basal metabolic rate (BMR) yielded comparable results to Model 1; energy intake adjustment yielded similar results to model 1 (OR = 1.45, 95% CI: 1.14, 1.83), whether or not BMR was also included in the model. Defining high-intake consumers by top quintile of sugar intake from soda yielded attenuated results yet a slight improvement in precision (OR = 1.27, 95% CI: 1.04, 1.53).

Sensitivity analyses yielded robust results. Excluding subjects with macroalbuminuria had no appreciable effect on the estimates. When we fit a linear regression model with log-transformed urinary albumin as the outcome, we obtained results consistent with Model 1: consumers of 2 or more sugary soft drinks having on average 1.13 mg/L higher albumin concentration than consumers of 0–1 drinks (95% CI: 1.06, 1.21 mg/L). Interaction terms with 2+ sugary drinks were significant for gender (p = 0.03), and for overweight (p = 0.01) but not for obesity (p = 0.42). Furthermore, log-transformed urinary albumin concentration was 1.09 mg/L higher among drinkers of 2 or more sugary colas (95% CI: 1.01, 1.18 mg/L), and 1.23 mg/L higher among sugary non-cola drinkers (95% CI: 1.09, 1.40 mg/L), than among non-soda drinkers; neither diet colas nor non-colas were related to albuminuria. Modeling continuous eGFR as a function of the covariates used in Model 1 did not yield statistically significant association with sugary soda consumption (p = 0.586); there was also no association when Stage 3 CKD (defined by eGFR<60 ml/min/1.73 m^2^) was substituted as the dependent variable in model 1 (p = 0.347). When the CKD cutpoint was changed to <45 ml/min/1.73 m^2^, the odds ratio increased to 2.82 ((95%CI:1.63,4.89), indicating that there may be an association between sugary soda and more advanced CKD.

## Discussion

**Figure 4:**
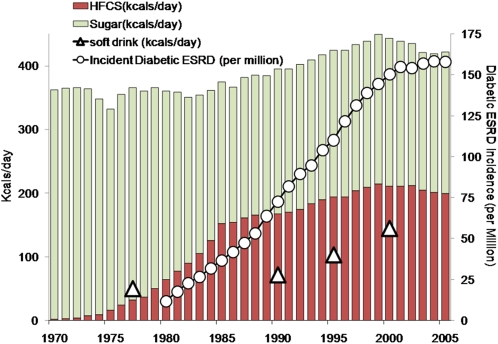
U.S. trends in total sugar availability, High Fructose Corn Syrup (HFCS) availability, soft drink consumption, and incident diabetic end-stage renal disease (ESRD) over time. Data on sweetener availability from USDA [38]; soft drink consumption from Nielsen and Popkin [8]; and incident diabetic ESRD from the United States Renal Data System [54].

We have found an association between sugar-sweetened sodas and albuminuria, which is a marker of early kidney damage. To our knowledge, this is the first report of such an association. In the United States, sugary sodas are predominantly sweetened with high fructose corn syrup [9], [37]. Over the past 30 years, both the availability of HFCS [38] and sugary soft drink consumption [8] have risen markedly. The prevalences of obesity, diabetes, and ESRD have all followed these trends; Figure 4 shows the temporal correlation of ESRD due to diabetes with calories due to high fructose corn syrup and soda consumption [6], [7]. While ESRD rates prior to 1992 may have risen due to better recognition and expanding treatment by renal replacement therapy, more recent trends likely reflect genuine increases in kidney failure [1], [39]. Because there are many unobserved characteristics that also change over time, inferring causation from correlated trends is problematic. Nevertheless, the finding of an individual-level association between sugary soda consumption and albuminuria are consistent with the hypothesis that HFCS is contributing to the kidney disease epidemic [6], [7]. We note that neither diet soda nor moderate intake of one serving of sugary soda was associated with albuminuria in this study.

The strongest associations were seen among those with lower body weight and females. It is not clear why females should be more susceptible to sugary soft drinks than males. Women were much less likely to consume 2 or more sugary drinks per day, eliminating residual confounding as an explanation. Women are also more likely to under-report (and men more likely to over-report) their energy intake according to perceived social norms [40]. Such misclassification may differentially bias results toward the null, because the absolute degree of under- or over-reporting differs by gender. Biological differences, such as lower energy intake need among women, might also account for the interaction of consumption and gender. However, this interaction was not an artifact of using gender-specific albuminuria cutpoints, because when we used log urinary albumin as the dependent variable, we still found significant interaction between gender and sugary soda consumption.

Optimal body weight may increase susceptibility because obesity is a competing mechanism by which sugar consumption could cause kidney damage [3]. If subjects are already obese, and their albuminuria is due to obesity, then any additional effects of sugary sodas would be attenuated. Alternatively, differential reporting may account for the apparent lack of association among the overweight and obese, as food intake is more likely to be underreported as BMI increases and body image satisfaction decreases [31], [32]. Nevertheless, underreporting by BMI is not supported by these data, as evidenced by the lack of a significant association between BMI categories and sugary soda consumption within quartiles of energy intake (Table 1).

Our results suggest that fructose may be nephrotoxic via pathways other than diabetes, long-term blood glucose level, hypertension, or obesity, because adjusting for these potential mediators did not eliminate the association. We caution that mediator-adjusted models are not causal models, and they rarely meet the conditions necessary to accurately identify independent effects of the main exposure [41]. Nevertheless, were these factors to truly explain our findings, we should have seen greater attenuation of the odds ratio than we did. It may be that fructose causes both kidney damage and obesity in parallel. Although fructose has not been directly linked to kidney disease, Johnson and Nielson have speculated that it may be directly pathogenic to human kidneys [6], [7]. The results are consistent with laboratory studies implicating fructose in rat models of kidney disease [42], [43], [44]. Johnson hypothesized that fructose – kidney associations may be explained by uric acid [6]. However, adjustment for uricemia did not attenuate this association in our study. Identification of pathways leading from albuminuria to kidney damage would strengthen the case for the observed association being a causal one.

Recent reports have shown that fructose consumption in the form of soft drinks increases the risk of kidney stones and gout [45], [46], which suggest a link between soda consumption, high fructose corn syrup, and chronic kidney disease. Diet sodas have also been associated with the development of metabolic syndrome [47], [48], which is itself a risk factor for CKD, although we did not find an association. We know of just one prior study directly examining soda consumption and kidney disease. Saldana and colleagues conducted a case-control study of 465 newly diagnosed chronic kidney disease patients in North Carolina, comparing their carbonated beverage consumption to 467 community controls [20]. They found an association between sugar-sweetened and diet colas with CKD, but not non-cola carbonated beverages. Our study differs from theirs in several respects. First, we used albuminuria as the outcome variable rather than diagnosed CKD. Albuminuria is a subclinical condition that may appear years before diagnosed CKD, and CKD may follow pathophysiological pathways that do not involve albuminuria. Second, the focus of Saldana's analysis was on carbonated beverages and cola consumption, not sugary soft drinks. They found similar odds ratios of sugar-sweetened and diet colas with CKD, while we found an association only with sugar-sweetened sodas, but not diet colas or other diet sodas. Third, they asked subjects to recall average adult beverage consumption prior to 1980, while NHANES was a cross sectional study that employed a 24-hour recall instrument. Studies relying on retrospective recall may yield biased results [49], although Saldana and colleagues discount the possibility of differential recall because data were collected before researchers suspected cola consumption might be linked to kidney disease. Another potential reason for the differences in findings is that carbonated beverages were overwhelmingly sweetened with sucrose in the early 1980s [50], perhaps accounting for Saldana's finding of similar associations between sugar-sweetened and diet cola beverages; in contrast, HFCS was in wide use when our study was conducted (see Figure 4) [38]. Finally, our study used nationally representative data, while theirs was confined to hospital patients and community controls in North Carolina.

Several limitations must be noted. First, this study was cross-sectional, precluding us from observing longitudinal associations. In particular, we could not determine if the complex interaction between BMI, soda consumption, and albuminuria was confounded by processes such as wasting associated with chronic disease [51]. Second, the NHANES surveys employ a 24 hour dietary recall and did not have measurement of diet over time, which may have led to underreporting of intake by obesity status. However, 24 hour recall provides valid estimates of group-average dietary intake [30]. Third, we did not have a direct measurement of intake of HFCS, which is ubiquitous in the American diet. If HFCS is the salient exposure, and non-consumers of sugary sodas have significant intake of HFCS from other sources, then we may have misclassified people by using soda consumption, underestimating the true effect. Fourth, albuminuria was assessed using a single specimen. In a random population-based sample (including non-diabetics), microalbuminuria persist in only 61% of participants with a single positive result [52]. Nevertheless, albuminuria persistence should be non-differential with respect to soda consumption, making the results reported here underestimates. Finally, tastes serve as markers of social class [53], suggesting that soda consumption is linked to myriad lifestyle factors that have not been fully captured here. This leaves open the possibility that these results are due to residual confounding, a problem generic to nutritional epidemiology studies. Understanding why sugary soda drinkers consume these beverages, while others do not, would improve both control of confounding, and potentially lead to more successful interventions.

In spite of these limitations, several strengths deserve mention. First, we used nationally representative data drawn from the NHANES surveys, yielding population-based measures of association that are generalizable to the general population, including racial and ethnic minorities. Second, because we had over 9,000 subjects and over 1,000 cases, we could explore effect measure modification (interactions) and estimate associations within subgroups of participants. Third, the richness of the NHANES dataset allowed us to adjust for a host of potential confounders and mediators, and associations with sugary soda consumption remained elevated even after adjusting for these factors. We conducted several sensitivity analyses, including modeling the log of albumin excretion, use of energy intake to define soda consumption, and adjustment for overall energy intake and basal metabolic rate. The associations were robust to different definitions of exposure, outcome, and adjustment factors. Finally, we found that the association of sugary soda consumption with eGFR below <45 ml/min/1.73 m^2^ was stronger than the association with albuminuria, which may reflect an association with more advanced chronic kidney disease [35]

In conclusion, we have found that sugary soft drink consumption is associated with albuminuria. While these results are consistent with prior knowledge, they can only suggest that HFCS plays a causal role in kidney disease. Longitudinal studies, with measures of HFCS and other sugar consumption, are needed to formally test this hypothesis. At this point in time, policy recommendations regarding soda consumption or HFCS would be premature. Our findings should be seen in the context of disparate associations with CKD and metabolic syndrome that have recently been found for colas (but not sugary non-colas) and diet sodas [20], [47], [48]. Additional study is needed before we may determine whether these findings are due to unmeasured lifestyle factors, other residual confounders, or truly causal associations.
